# Activation of RhoA, but Not Rac1, Mediates Early Stages of S1P-Induced Endothelial Barrier Enhancement

**DOI:** 10.1371/journal.pone.0155490

**Published:** 2016-05-17

**Authors:** Xun E. Zhang, Shaquria P. Adderley, Jerome W. Breslin

**Affiliations:** Department of Molecular Pharmacology and Physiology, Morsani College of Medicine, University of South Florida, Tampa, Florida, United States of America; Texas A&M University Health Science Center College of Medicine & Baylor Scott and White Health, UNITED STATES

## Abstract

Compromised endothelial barrier function is a hallmark of inflammation. Rho family GTPases are critical in regulating endothelial barrier function, yet their precise roles, particularly in sphingosine-1-phosphate (S1P)-induced endothelial barrier enhancement, remain elusive. Confluent cultures of human umbilical vein endothelial cells (HUVEC) or human dermal microvascular endothelial cells (HDMEC) were used to model the endothelial barrier. Barrier function was assessed by determining the transendothelial electrical resistance (TER) using an electrical cell-substrate impedance sensor (ECIS). The roles of Rac1 and RhoA were tested in S1P-induced barrier enhancement. The results show that pharmacologic inhibition of Rac1 with Z62954982 failed to block S1P-induced barrier enhancement. Likewise, expression of a dominant negative form of Rac1, or knockdown of native Rac1 with siRNA, failed to block S1P-induced elevations in TER. In contrast, blockade of RhoA with the combination of the inhibitors Rhosin and Y16 significantly reduced S1P-induced increases in TER. Assessment of RhoA activation in real time using a fluorescence resonance energy transfer (FRET) biosensor showed that S1P increased RhoA activation primarily at the edges of cells, near junctions. This was complemented by myosin light chain-2 phosphorylation at cell edges, and increased F-actin and vinculin near intercellular junctions, which could all be blocked with pharmacologic inhibition of RhoA. The results suggest that S1P causes activation of RhoA at the cell periphery, stimulating local activation of the actin cytoskeleton and focal adhesions, and resulting in endothelial barrier enhancement. S1P-induced Rac1 activation, however, does not appear to have a significant role in this process.

## Introduction

The endothelial cells of capillaries and postcapillary venules form a semi-permeable barrier that is crucial for normal blood-tissue exchange and tissue homeostasis. Compromised endothelial barrier function which occurs during inflammation [1] significantly contributes to a wide variety of pathologies, including the systemic inflammatory response syndrome [2], ischemia-reperfusion injury [3,4], atherosclerosis [5], and cancer cell metastasis [6]. The mechanisms that control endothelial barrier function have long been a key focus of investigation, yet remain incompletely understood.

Continuous cytoskeleton maintenance is critical for normal endothelial barrier function [7,8], and significant cytoskeletal rearrangements often accompany changes in permeability. For example, actin stress fibers are typically elicited by inflammatory agents that compromise barrier function [9]. In contrast, strengthening of cortical actin at the cell periphery has been postulated to enhance endothelial barrier function [10,11]. In addition, we recently reported that dynamic changes in the normal cycling of actin-rich local lamellipodia in endothelial cells correlated with alterations in barrier function [12–14].

Rho family small GTPases strongly influence the actin cytoskeleton and have been shown to be important for controlling endothelial barrier integrity. Several studies have shown that RhoA activation correlates with increased permeability of the endothelium [13,15,16]. In contrast, activation of Rac1 has been correlated with endothelial barrier enhancement [12,17,18]. Collectively these data have led to the general notion that Rac1 activation enhances endothelial barrier enhancement, while RhoA activation disrupts integrity of the endothelium [19]. Some recent data have challenged to this paradigm, however. An elegant study by Szulcek and colleagues demonstrated that RhoA activation at the cell periphery correlates with barrier integrity, while its activation in the perinuclear area of the endothelial cell contributes to barrier disruption [20]. Another observation challenging this paradigm is our recent finding that sphingosine-1-phosphate (S1P) elicits a strong increase in the GTP-bound, activated forms of both RhoA and Rac1 [12]. In addition, Xu and colleagues previously observed that inhibition of RhoA’s downstream mediator, Rho kinase (ROCK), attenuates S1P-induced endothelial barrier enhancement [21]. These findings raise the question about the relative involvement of RhoA and Rac1 in S1P-induced endothelial barrier enhancement, and whether spatiotemporal activation of RhoA is a key factor.

For this study we focused on the endothelial barrier enhancement elicited by S1P, an endogenously released, bioactive lipid that is a potent endothelial barrier enhancer at its physiological concentration [22,23]. After binding to its receptors, S1P induces dynamic cytoskeletal, junctional and adhesion changes and decreases permeability [24]. In our previous observation of S1P-induced activation of both Rac1 and RhoA, the Rac1 activation was relatively short lived, while RhoA activation was strong and sustained [12]. In addition, we and other groups have reported that S1P causes cortical Myosin light chain-2 (MLC-2) phosphorylation [10–12,25], which is thought to help stabilize barrier integrity [17,26]. It is unknown to what extent elevated RhoA activity is responsible for S1P-induced MLC-2 phosphorylation. In the current study, we investigated the roles of Rac1 and RhoA in S1P-induced endothelial barrier enhancement, the spatiotemporal activation of RhoA in response to S1P, and the potential roles MLC2 phosphorylation, actin fiber formation, and localization of the focal adhesion protein vinculin.

## Materials and Methods

### Materials

Human umbilical vein endothelial cells (HUVEC), human adult dermal microvascular endothelial cells (HDMEC), Endothelial Growth Medium-2MV (EGM2-MV), and Endothelial Basal Medium (EBM) were all purchased from Lonza (Basel, Switzerland). The Ingenio^®^ electroporation kit and solution were obtained from Mirus Bio LLC (Madison, WI). The pcDNA3-GFP-Rac1 (wild type; WT) and pcDNA3-GFP-Rac1T17N (dominant negative; DN) plasmids were purchased from Cell Biolabs (San Diego, CA). Rac1 siRNA (Knockdown: UAAGGAGAUUGGUGCUGUA) and control RNA (Non-Targeting: UGGUUUACAUGUCGACUAA) were purchased from Thermo Scientific (Rockford, IL); pTriEx-RhoA FLARE.sc Biosensor WT [27] was a gift from Klaus Hahn (Addgene plasmid #12150). Rac1 Inhibitor Z62954982, and RhoA inhibitors Y16 and Rhosin, were purchased from Merck-Millipore (Billerica, MA). Spingosine-1-phosphate was purchased from Tocris (Bristol, UK). All other drugs and chemicals were purchased from Sigma-Aldrich (St. Louis, MO). HRP-conjugated-Mouse anti-β-actin (sc-47778 HRP) was purchased from Santa Cruz Biotechnology, Inc. (Santa Cruz, CA). Rabbit anti phospho-MLC2-T18/S19 (#3674) was purchased from Cell Signaling Technology (Boston, MA). Mouse anti vinculin (ab18058) was purchased from Abcam (Cambridge, UK). Alexa Fluor 488-donkey anti-rabbit IgG antibody (A21206) and 488-donkey anti-mouse IgG antibody (A21202) were purchased from Invitrogen (Carlsbad, CA).

### Cell Culture and Transfection

HUVEC and HDMEC were routinely grown in EGM2-MV in 1.5% gelatin-coated culture dishes. For all studies, passage 1–5 cells were used. For transfection, cells grown to 80% confluence were trypsinized and pelleted, and 5 X 10^5^ cells were resuspended in 100 μl electroporation master mix containing either 2 μg plasmids or 2 μM siRNA. This mixture was transferred to a cuvette for transfection using a Nucleofector II system (Lonza, Basel, Switzerland) with program A-034 (HUVEC) or M-003 (HDMEC). Warm EGM2-MV (500 μl) was added into the cuvette immediately after electroporation. Cells were later distributed evenly onto gelatin-coated 35-mm dishes for protein extraction, gelatin-coated MatTek 35-mm #1 glass bottom dishes for time-lapse microscopy, or 96W1E ECIS arrays (Applied Biophysics, Troy, NY) for determination of barrier function.

### Immunoblotting

Cell protein lysates were obtained as previously described [12,28]. Protein levels were quantified with the BCA protein assay kit (Thermo Scientific, Rockford, IL). Protein (15 μg) was mixed with NuPAGE^®^ Reducing agent containing NuPAGE^®^ LDS sample buffer (Invitrogen, Carlsbad, CA), heated at 70°C for 10 min, and loaded into Novex^®^ 4–12% Bis-Tris precast gels (Invitrogen, Carlsbad, CA) for SDS-PAGE. Proteins were transferred onto 0.45 μm PVDF membrane and blocked with 5% BSA in TBST (20mM Tris-HCl, PH 7.6, 150mM NaCl, 0.1% Tween 20). Membranes were incubated with primary antibodies at 4°C overnight, and washed three times in TBST. Afterwards, secondary antibodies were applied at room temperature for 1 h, followed by three washes with TBST. Bands were visualized with Supersignal^®^ HRP substrate (Thermo Scientific, Rockford, IL) and imaged with Bio-Rad Gel Doc™ system (Bio-Rad, Hercules, CA).

### Endothelial Barrier Function

Transendothelial electrical resistance (TER), which serves as an index of barrier function of cultured endothelial cell monolayers, was determined with an Electrical Cell-Substrate Impedance Sensor (ECIS) ΖΘ System (Applied Biophysics, Troy, NY). Cells were seeded into gelatin-coated wells of ECIS arrays (96W1E) and allowed to attach overnight in EGM2-MV to form a confluent monolayer. The next day, medium was changed to EBM at least 1 h before the experiment. A 1-μA AC signal at 4 kHz was applied. Total impedance was reported by monitoring the voltage across the electrodes and its phase relative to the applied current. The cell-covered electrode unit was treated as an RC circuit, from which impedance data was later converted into monolayer resistance and capacitance, respectively representing barrier function and membrane capacitance [29].

### Analysis of RhoA Activation by FRET

Cells were transfected with the pTriEx-RhoA FLARE.sc biosensor and seeded onto 1.5% gelatin coated MatTek 35 mm #1 glass bottom dishes (MatTek Corp., Ashland, MA), and grown overnight to confluence. The medium was changed to EBM 3 h before the experiment. Each MatTek plate containing cells was transferred to a temperature-controlled (37°C) imaging chamber. Time-lapse imaging data was acquired with a Leica SP2 confocal microscope with 63X objective at 30-s intervals, using the YFP and CFP channels (Leica Microsystems, Buffalo Grove, IL) in the USF Lisa Muma Weitz Laboratory for Advanced Microscopy and Cell Imaging. Briefly, images were first cropped, background subtracted and converted to 32-bit images. Further processing was done by smoothing both channels, threshold the FRET channel and finally convert the images to ratio images alter we converted (FRET/CFP) by using the analysis method described by Kardash et al [30] Images were saved as TIFF for presentation.

### Immunofluorescence Confocal Microscopy

Immunofluorescence labeling and confocal microscopy was performed as previously described [12,28]. Briefly, cells were fixed with 4% paraformaldehyde and permeabilized with 0.1% Triton X-100 in PBS. The cells were blocked with 10% donkey serum in PBS, incubated with primary antibodies at 4°C overnight, washed 3X in antibody wash buffer, incubated with secondary antibodies at room temperature for 1 h, and washed 3X again. The cells were then incubated with Texas Red-phalloidin at room temperature for 30 min. The slides were mounted with ProLong Gold antifade reagent containing DAPI to label the nuclei (Life Technologies, cat. no. P36931). Confocal Images were acquired with Olympus FV1000 microscope system by using a 60X oil immersion objective (Olympus America, Center Valley, PA) in the USF Lisa Muma Weitz Laboratory for Advanced Microscopy and Cell Imaging.

### Data Analysis

All data are shown as mean ± SE. For two group comparisons, student t-tests were used. For comparisons of 3 or more groups, one-way ANOVA was used, with Tukey's multiple comparisons test for post-hoc analysis. For multiple group comparisons over time, repeated measures two-way ANOVA followed by Tukey's multiple comparisons test was used. Significance was accepted as P<0.05.

## Results

### Rac1 is important for baseline barrier integrity

**Fig 1:**
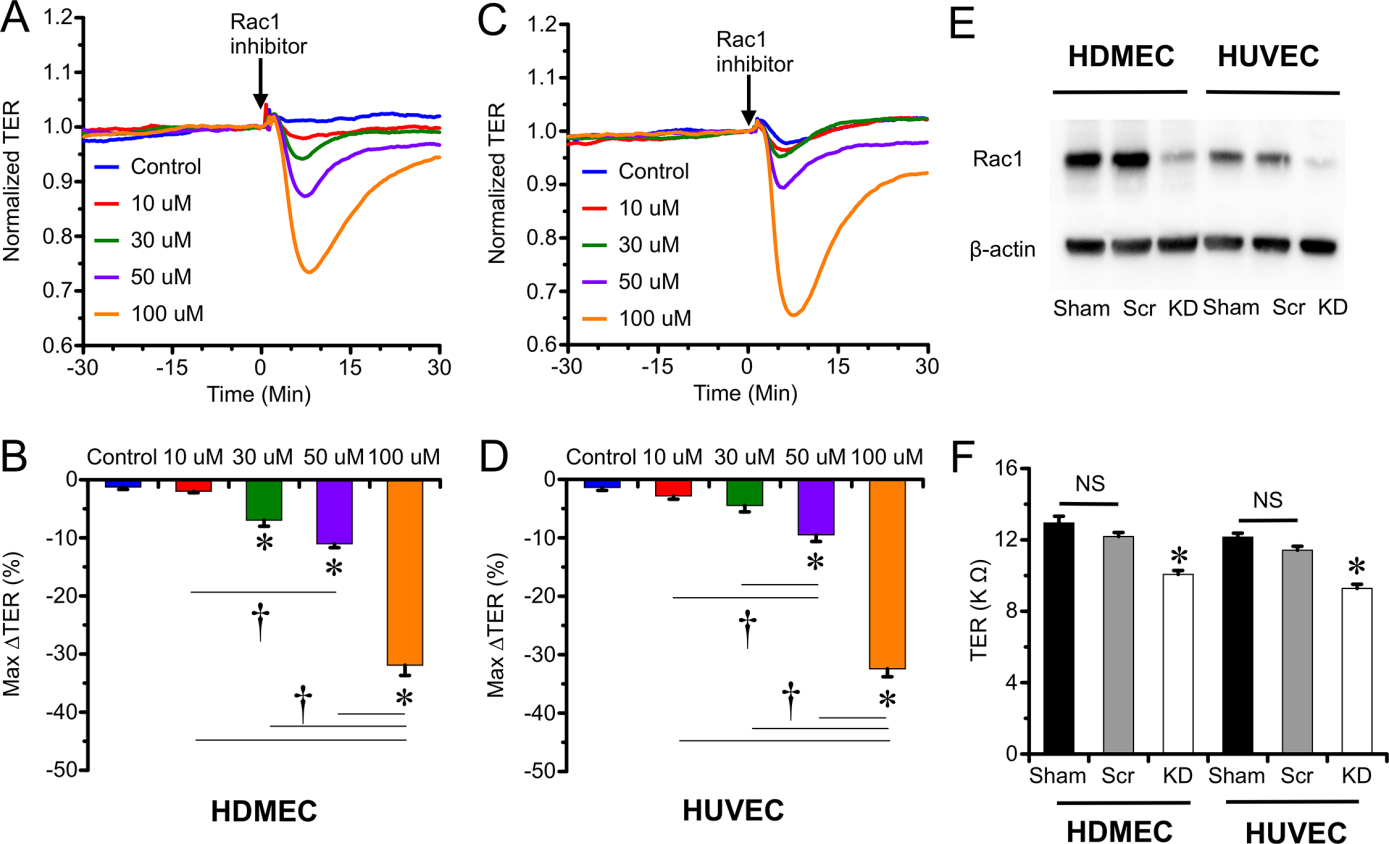
Pharmacological inhibition or siRNA-mediated knockdown of Rac1 impaired baseline endothelial barrier integrity. *A*. Treatment with the selective Rac1 inhibitor Z62954982 reduces TER in a concentration-dependent manner in HDMEC monolayers. *B*. Comparison of the mean maximum decreases in TER in the 30-min time window for each concentration of Z62954982 in HDMEC monolayers. Panels *C & D* show that Z62954982 produces a similar concentration-dependent TER in HUVEC monolayers. *E*. Western blot confirming knockdown (KD) with Rac1-specific siRNA, compared to sham and scrambled RNA (Scr) control groups. Bands for β-actin from re-probed blots confirmed equivalent loading of protein for each lane. *F*. Mean baseline TER values of HDMEC and HUVEC monolayers in Rac1 knockdown, scrambled control, and sham-transfected groups. *P<0.05 versus vehicle treated group. †P<0.05 versus other concentrations.

We previously observed that overexpression of Rac1 can reduce permeability of endothelial monolayers [12]. Here, we tested whether Rac1 is critical for maintaining baseline barrier integrity in both HUVEC and HDMEC monolayers. Treatment with the Rac1 inhibitor Z62954982 caused a concentration-dependent decrease in TER in both HDMEC (Fig 1A & 1B) and HUVEC (Fig 1C & 1D). We also used a second approach, which was to knock down Rac1 expression using siRNA. Significant Rac1 knockdown was achieved in both HUVEC and HDMEC as assessed by Western blot (Fig 1E). Rac1 knockdown rendered a significantly lower baseline TER at 72 h compared to controls (Fig 1F). Taken together, our data indicate that Rac1 has a critical role in the maintenance of baseline endothelial barrier integrity.

### S1P rapidly increased endothelial barrier function in concentration-dependent manner

**Fig 2:**
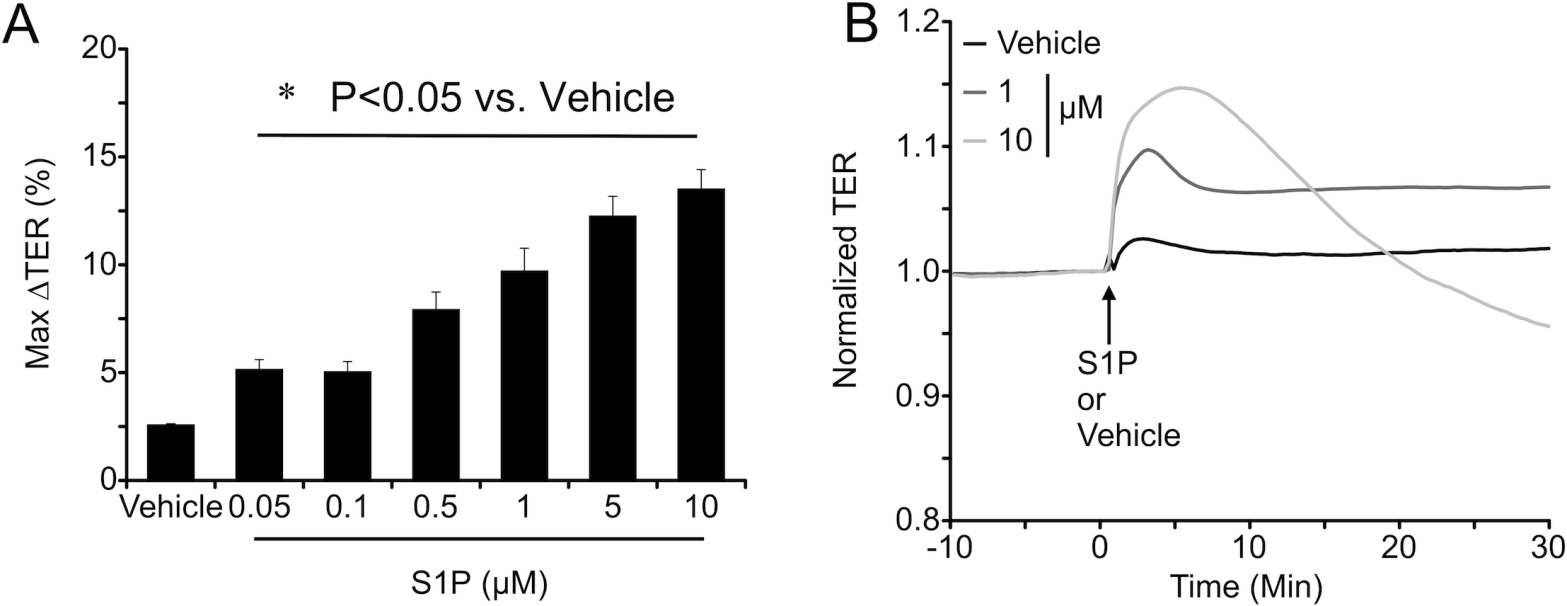
S1P caused initial endothelial barrier enhancement is concentration-dependent manner. *A*. Maximal change in TER (%) from baseline within the initial 10-min time window after addition of the indicated concentrations of S1P. *P<0.05 versus vehicle treated group. *B*. Comparison of representative tracings of TER of HUVEC monolayers treated with 1 μM or 10 μM S1P. After the initial increase in TER, with higher concentrations of S1P (10 μM shown here), this elevated TER is typically short-lived and often decreases to a level below the initial baseline.

S1P has a well-known role in the enhancement of endothelial barrier function, although recently it has been reported that high concentrations of S1P can disrupt endothelial barrier integrity [31]. Therefore, we performed a concentration-response study with S1P on HUVEC monolayer barrier function (Fig 2). When examining the maximum increase in TER that could be elicited by each concentration of S1P, we observed that treatment with S1P significantly increased TER compared to vehicle control in a concentration-dependent manner (Fig 2A). However, it is worth noting that while all concentrations tested caused a rapid, significant, initial rise in TER, with higher concentrations of S1P this elevation in TER was typically not sustained, and sometimes fell below the baseline TER within 30 min (Fig 2B).

### Inhibition of Rac1 failed to block S1P-induced endothelial barrier enhancement

**Fig 3:**
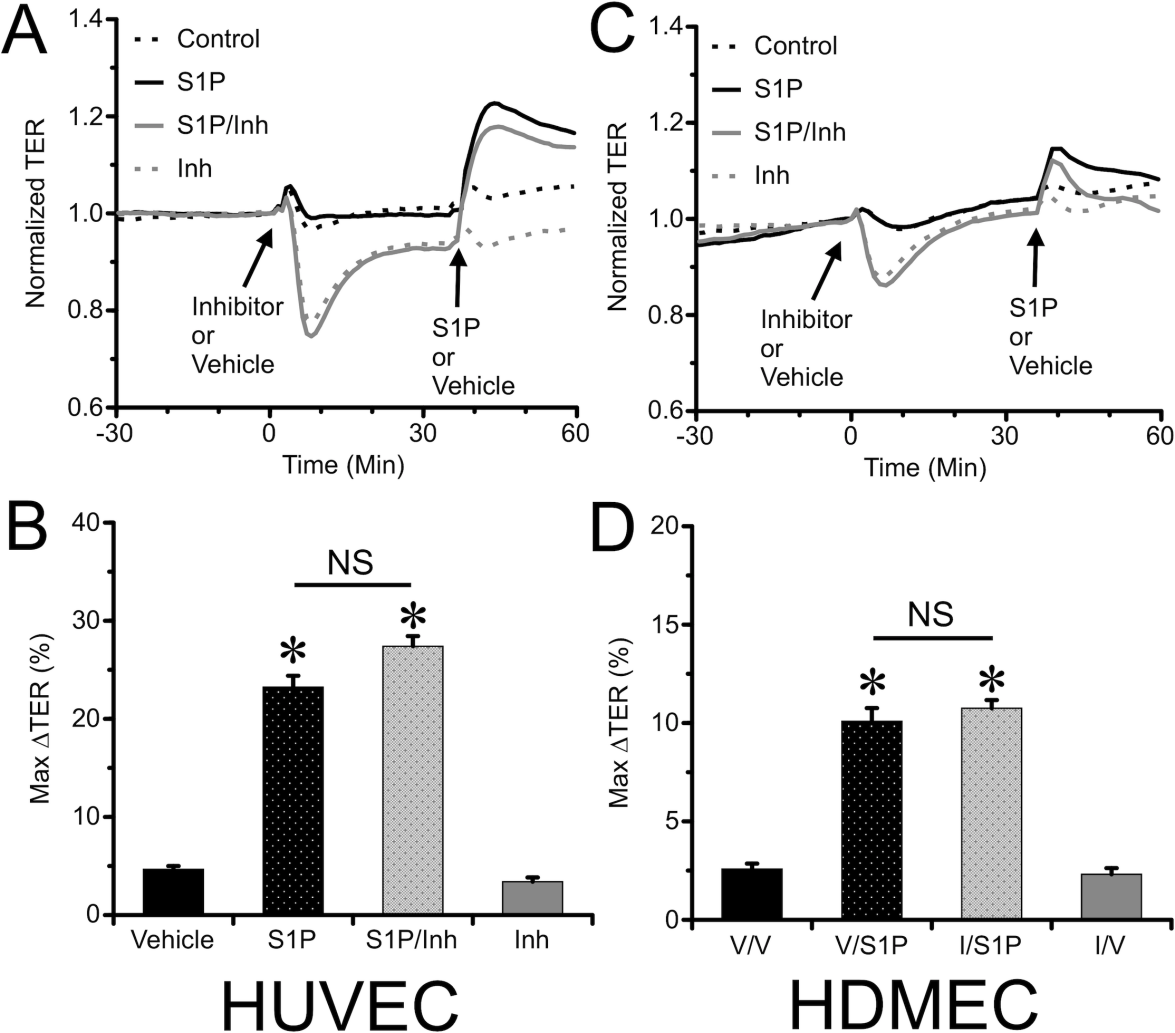
Pharmacologic inhibition of Rac1 failed to block S1P-induced endothelial barrier enhancement in HUVEC and HDMEC monolayers. *A*. The time course of changes in of TER of HUVEC monolayers pretreated with the 30 min with Rac1 inhibitor Z62954982 or vehicle control, followed by treatment with 2 μM S1P are shown (N = 8 for each group). *B*. Mean maximal change in TER (%) of HUVEC monolayers after S1P treatment within the first 10-min window. Panels *C & D* show corresponding results for HDMEC monolayers (N = 8 each group). *P<0.05 vs. S1P Vehicle pretreated groups.

Rac1 has been previously reported to mediate the barrier protective effect of S1P [23]. We tested the extent to which the selective Rac1 inhibitor Z62954982 would attenuate S1P-induced endothelial barrier enhancement. We applied the inhibitor at a concentration of 50 μM (based on our results in Fig 1) to either HUVEC or HDMEC (Fig 3A & 3C). To our surprise, after pretreatment with Z62954982, S1P still significantly increased TER in a similar manner as with cells that did not receive pretreatment with the inhibitors (Fig 3B & 3D). These data suggest that the endothelial barrier enhancement elicited by S1P may not require Rac1 activation.

### Overexpression of WT Rac1 or DN Rac1 did not affect S1P-induced barrier enhancement

**Fig 4:**
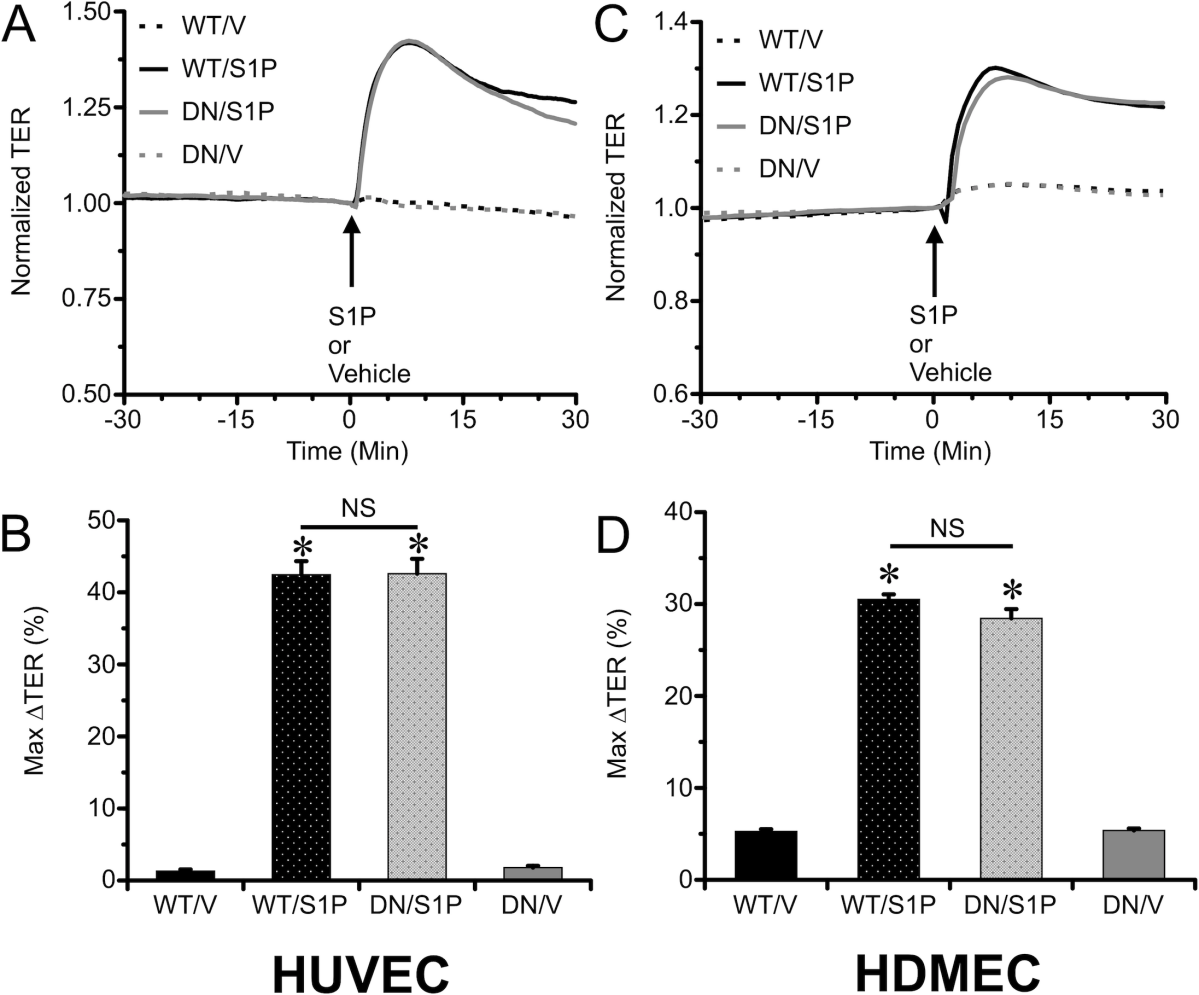
Overexpression of WT or DN Rac1 in HUVEC or HDMEC did not alter S1P-induced endothelial barrier enhancement. *A*. Time course of TER of HUVEC monolayers transfected with WT and DN Rac1 plasmids, treated with either 2 μM S1P or vehicle (N = 8 each group). The TER is normalized to the time point just prior to addition of S1P for a more direct comparison of the magnitude of the response. *B*. The mean maximal change in TER (%) of HUVEC monolayers in the 10-min window immediately following S1P treatment. The corresponding data for HDMEC monolayers are shown in panels *C & D* (N = 8 each group). *P<0.05 vs. Vehicle treated groups.

A second approach we used to test the role of Rac1 was to transfect HUVEC or HMDEC with WT and DN Rac1 plasmids (Fig 4), which we previously showed can modulate baseline barrier function [12]. However, again to our surprise, S1P treatment significantly increased TER in the cells transfected with DN Rac1 in a similar manner to WT Rac1 (Fig 4B & 4D). These data provide additional evidence that S1P is able to enhance endothelial barrier function independently of Rac1 activation.

### Knockdown of Rac1 with siRNA did not affect S1P-induced endothelial barrier enhancement

**Fig 5:**
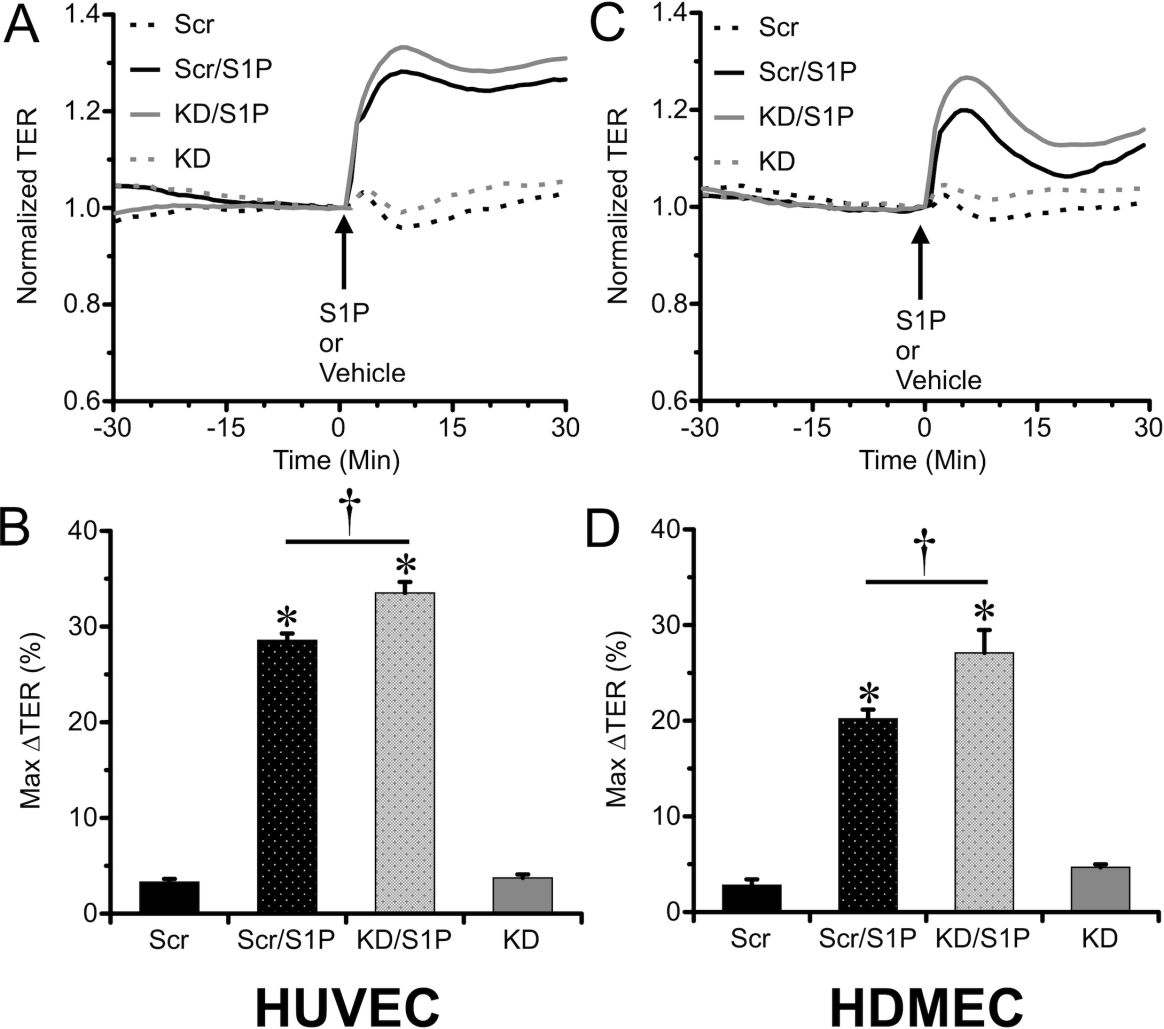
Knockdown of Rac1 expression with siRNA did not diminish S1P-induced barrier enhancement of HUVEC or HDMEC monolayers. A. Time course of changes in TER of HUVEC monolayers before and after treatment with 2 μM S1P or vehicle control, for the Rac1 knockdown and scrambled RNA transfected groups (N = 8 each group). The TER is normalized to the time point just prior to the addition of S1P, for more direct comparisons of the responses to S1P between the groups. *B*. The mean maximal change in TER of HUVEC monolayers (%) during the first 10 min after S1P was added. The corresponding results for HDMEC monolayers are shown in panels *C & D* (N = 8 each group). *P<0.05 vs. vehicle control groups; †P<0.05 vs. scrambled RNA sequence group.

A third approach we used was to test the extent to which siRNA-induced Rac1 knockdown would inhibit S1P-induced endothelial barrier enhancement (Fig 5). Despite the significant reduction in Rac1 expression and reduction in TER (Fig 1F), no inhibitions of S1P-induced increases in TER were apparent (Fig 5A & 5C). To our surprise, the S1P-induced elevation in TER was significantly increased in the Rac1 knockdown HUVEC and HDMEC, compared to transfection with the Scrambled RNA (Fig 5B & 5D), probably because the baseline TER is slightly lower after Rac1 knockdown (see Fig 1F; The TER data in Fig 5 are normalized to the time point just prior to addition of S1P). These data demonstrate that reduction of Rac1 expression does not impair the ability of S1P to enhance endothelial barrier function.

### Inhibition of RhoA attenuated S1P-induced barrier enhancement

**Fig 6:**
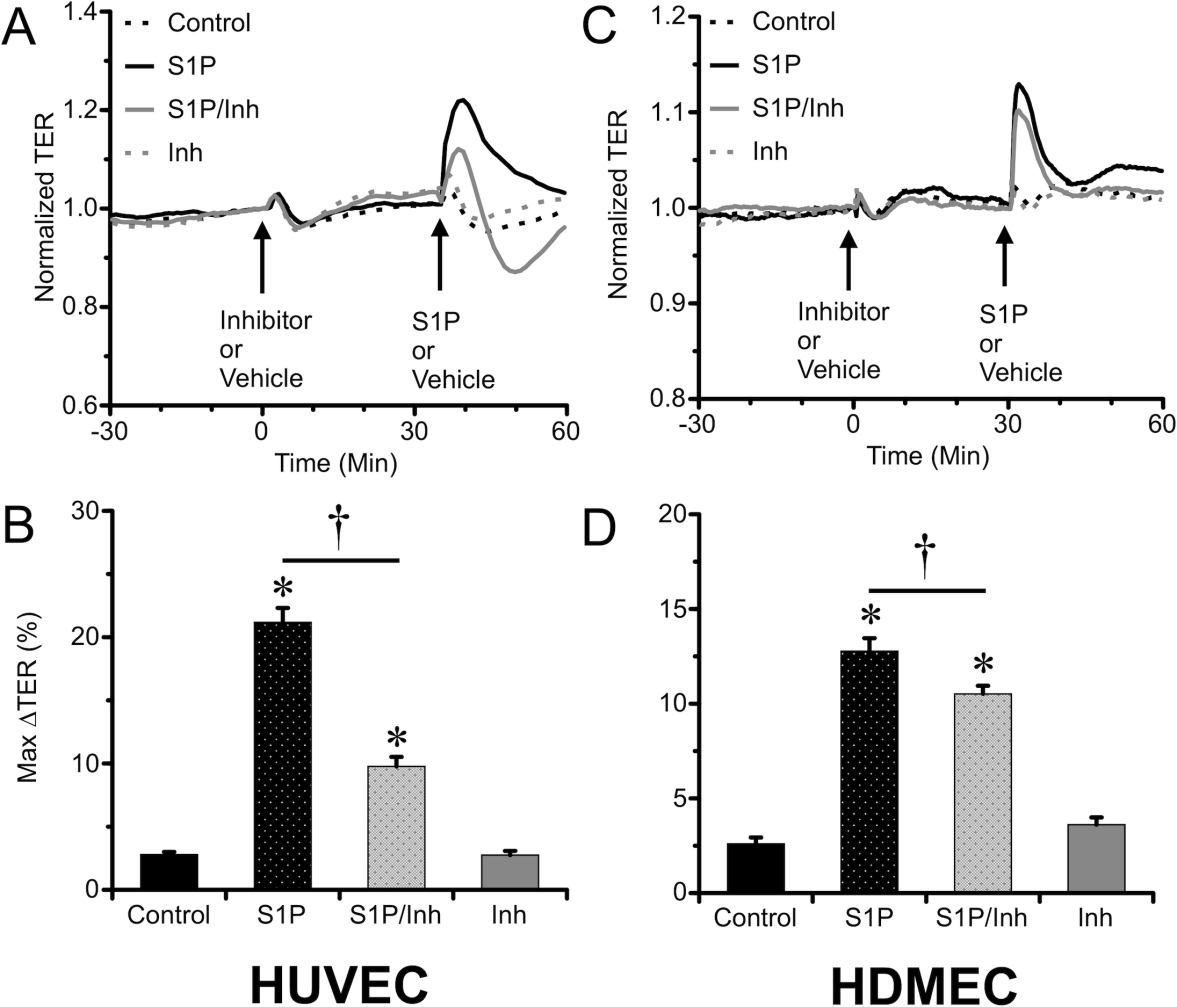
Inhibition of RhoA attenuated S1P-induced barrier enhancement of endothelial monolayers. *A*. The time course of changes in of TER of HUVEC monolayers pretreated with the 30 min with the combination of Rhosin and Y16 (5 μM of each) or vehicle control, followed by treatment with 2 μM S1P or vehicle (N = 8 for each group). *B*. Comparison of the mean maximal changes in TER of HUVEC monolayers (%) within the first 10 min after S1P or vehicle. The corresponding results for HDMEC monolayers are shown in panels *C & D* (N = 8 each group). *P<0.05, S1P vs. vehicle treated group. †P<0.05, inhibitor vs. vehicle pretreatments.

We previously reported that that S1P causes a strong and sustained activation of RhoA in HUVEC [12]. To test whether inhibition of RhoA impacts the S1P-induced increase in TER, we utilized the specific RhoA inhibitors Rhosin (25 μM) and Y16 (25 μM) on both HUVEC and HDMEC. As the two drugs can work synergistically to keep RhoA in its inactive, GDP-bound form [32], we also utilized their combination (5 μM each). In HUVEC, pretreatment with either Rhosin or Y16 alone significantly reduced S1P-induced increase in TER, but each drug alone did not cause a significant inhibition in HDMEC (S1 and S2 Figs). However, in both HUVEC and HDMEC, pretreatment with the combination of Rhosin and Y16 caused a significant attenuation of the S1P-induced increase in TER (Fig 6). These data suggest that RhoA activation partially mediates S1P-induced endothelial barrier enhancement, although to different extents in HUVEC and HDMEC.

### S1P-induced RhoA activation occurred primarily at the endothelial cell periphery

**Fig 7:**
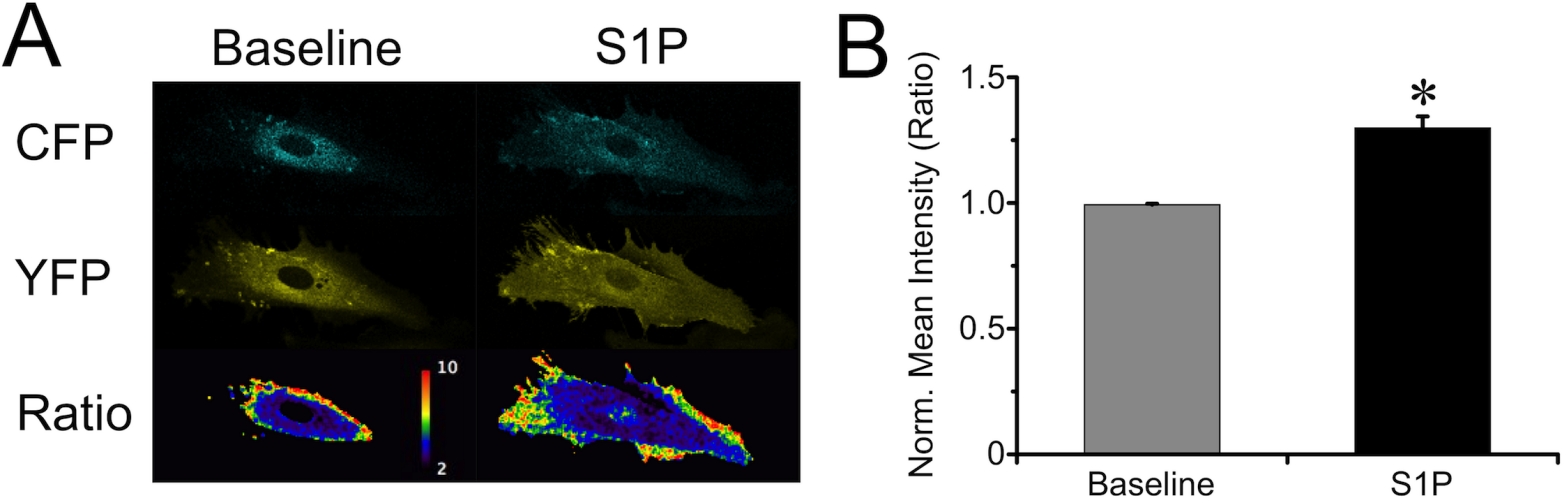
S1P activated RhoA primarily at cell periphery. *A*. Representative images of HUVEC expressing the pTriEx-RhoA FLARE.sc Biosensor, showing the CFP and YFP channels, and the ratio (FRET) indicating RhoA activation, during baseline and after the treatment of 2 μM S1P. The entire time course can be viewed in S1 Movie. *B*. Normalized mean intensity of the whole cell before and after S1P treatment (N = 5 cells studied). *P<0.05, before vs. after S1P treatment.

As the RhoA inhibitor produced its maximum inhibitory effect on HUVEC, we focused on HUVEC for further studies of the mechanism. The recent report of differential localized RhoA activation in endothelial cells during endothelial barrier maintenance and disruption [20] prompted us to investigate the localization of RhoA activation after stimulation with S1P. We transfected HUVEC with a RhoA FRET biosensor to measure RhoA activation over time in individual cells. During baseline, RhoA activation at any given point in the cell was low and oscillatory in nature, primarily located in the outer peri-nuclear region. S1P treatment significantly increased RhoA activity, shifting the maximal activity primarily near cell borders (Fig 7 and S1 Movie). The data suggest that S1P elicits a specific spatiotemporal activation of RhoA near the borders in order to promote enhanced endothelial barrier function.

### Inhibition of RhoA attenuated S1P-induced myosin light chain 2 (MLC-2) phosphorylation

**Fig 8:**
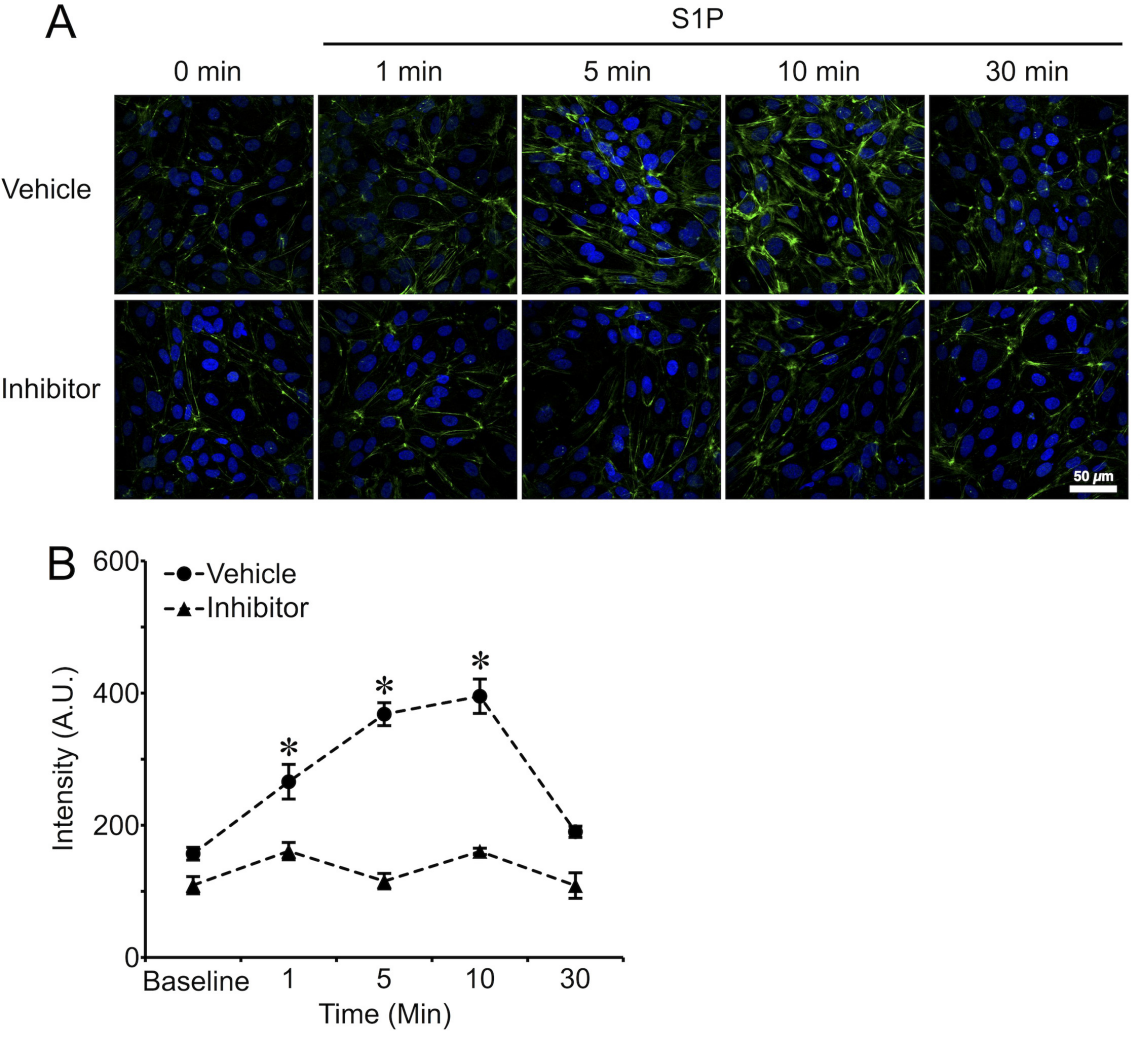
Inhibition of RhoA abrogated S1P-induced phosphorylation of MLC-2 on Thr-18/Ser-19 that is primarily near cell borders. A. Z-projection confocal immunofluorescence microscopy images of phosphorylated MLC-2 on HUVEC monolayers are shown. Each image represents three replicates for each time point. S1P was applied at 2 μM. Combined Rhosin and Y16 pretreatment was for 30 min, at 5 μM each. B. Quantification of phosphorylated MLC-2 intensity for each time point. *P<0.05, S1P treatment compared with baseline.

Because S1P elicited increased phosphorylation of MLC-2 in the vicinity of cortical actin near the cell junctions is thought to confer stronger endothelial barrier function [11], we evaluated the extent to which inhibition of RhoA with combined Rhosin and Y16 (both at 5 μM) can block S1P-induced MLC-2 phosphorylation on its Ser18/Thr19 activation site (Fig 8A). The results show that S1P rapidly increased MLC-2 phosphorylation within 1 min, demonstrating a peak at 10 min that returned to baseline level by 30 min. Inhibition of RhoA completely blocked this S1P-induced phosphorylation of MLC-2 (Fig 8B). These data suggest that RhoA is a critical mediator for S1P-induced phosphorylation of MLC-2.

### Inhibition of RhoA attenuated S1P-induced Vinculin mobilization to the cell periphery

**Fig 9:**
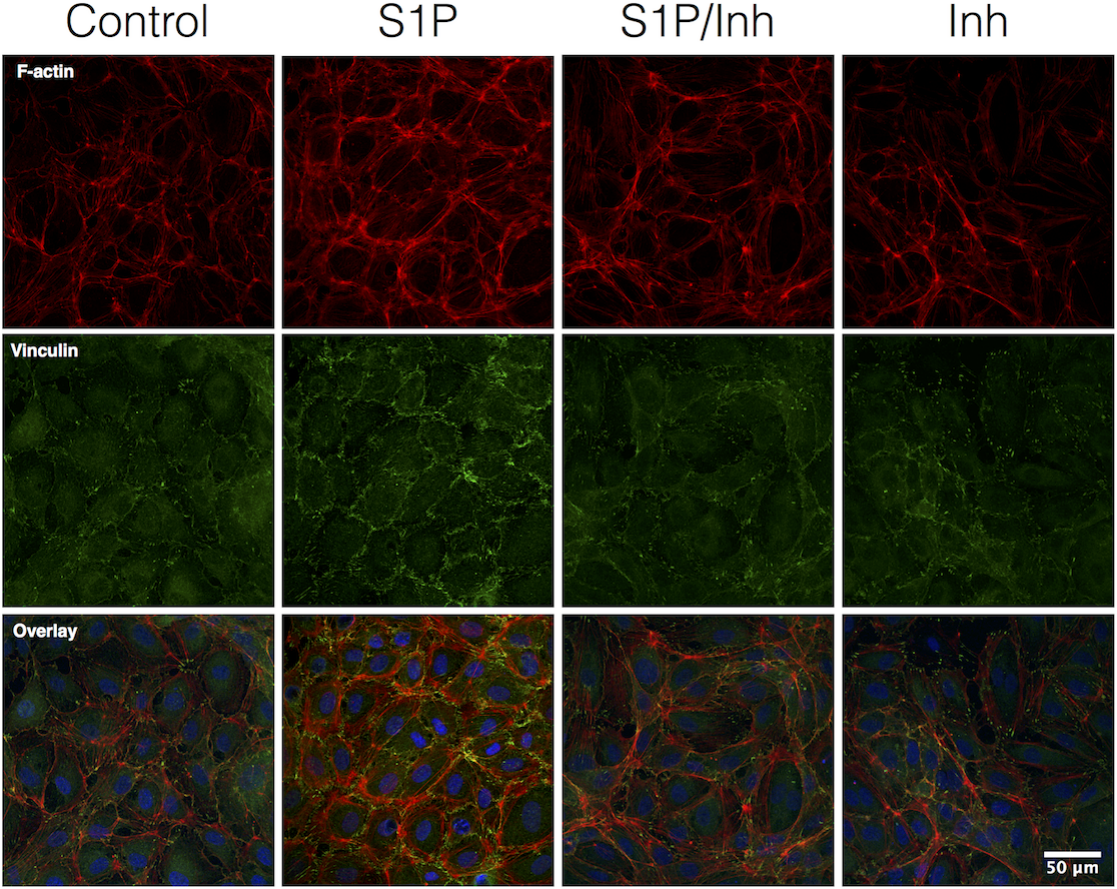
RhoA inhibition abrogated S1P-induced F-actin formation and recruitment of vinculin near the cell periphery. The results showed that S1P increases F-actin and vinculin labeling in the peripheral areas of cells (10 min after the addition of S1P). This was inhibited after pretreatment with combined Rhosin and Y-16 (Inh; 5 μM each, 30 min). The inhibitors alone had no impact. All images are representative of 3 separate experiments.

Both RhoA activation and MLC-2 phosphorylation has been implicated in the reorganization of the actin cytoskeleton and formation of focal adhesions [33]. We tested if the role of RhoA in the formation of actin fibers and vinculin-containing focal adhesions near intercellular junctions, Pretreatment with Rhosin and Y16 abrogated S1P-induced F-actin and vinculin recruitment to the cell periphery (Fig 9). These data suggest that S1P-induced F-actin and vinculin assembly at the cell periphery involves RhoA activation.

## Discussion

In this study, we present new evidence that Rac1 activation is not required for S1P to enhance endothelial barrier function in both HUVEC and HDMEC monolayers. We were initially surprised by these results based on the well-established role of Rac1 in maintaining baseline endothelial barrier function [17,18] and reports that have suggested its role in S1P-induced enhancement of the endothelial barrier [12,34]. However, closer investigation of the literature revealed that the data directly supporting the role of Rac1 in S1P-induced endothelial barrier enhancement were quite limited. To our knowledge there have been no previous investigations that rigorously coupled siRNA knockdown of Rac1, DN Rac1 expression, and pharmacologic strategies all in one model to test the role of Rac1 in S1P-induced endothelial barrier enhancement.

To understand discrepancies between our study and reports in the literature, it is important to discuss the time course of the endothelial barrier response to S1P. Our current results indicate that all concentrations of S1P tested (0.05–10 μM) initially increase barrier function of HUVEC monolayers (Fig 2A), but with higher concentrations of S1P (10 μM), this increase is often not sustained (Fig 2B). Previously it was reported that physiological concentrations (10 nM– 2 μM) of S1P enhance endothelial barrier function [35,36], but that high concentrations of S1P (≥ 5 μM) disrupt endothelial monolayer integrity [31,37], however detail of the time-course of changes in barrier function were limited. Adamson *et al* reported that 1 μM S1P decreased hydraulic conductivity of single-perfused microvessels at 30 min but not 60 min, while higher concentrations (5 and 10 μM) increased hydraulic conductivity at 60 min but not 30 min [37]. Other groups have reported similar results [15,31,38]. In our previous study, we showed that 2 μM S1P caused initial, rapid spreading and subsequent contraction of endothelial cells, and that the S1P-induced initial increase in TER directly correlated with increased protrusion of local lamellipodia at endothelial junctions [12]. It is clear that different S1P concentrations have distinct impacts on whether the initial endothelial barrier enhancement is sustained. While this study focused mainly on the mechanism responsible for the early stage endothelial barrier enhancement by S1P, a potential limitation is that higher concentrations of S1P may activate additional receptor subtypes and differentially affect Rho family small GTPases, or stimulate additional signals, resulting in a less sustained response and eventual reduction in barrier function, explaining the findings reported in other studies [31,37].

We confirmed that Rac1 is critical for baseline barrier integrity, as shown by other investigators [19,39]. However, we found no evidence that inhibition of Rac1 activation, whether by pharmacologic agents, overexpression of dominant-negative Rac1, or depletion of Rac1 with siRNA, could inhibit S1P-induced barrier enhancement. One previous report indicated that Rac1 depletion with siRNA ablated TER enhancement following 1 μM S1P treatment in HPAEC, but this data was limited to a single time point at 60 min [40]. Studies utilizing expression of a dominant-negative Rac1 in HPAEC showed reduction of the peak S1P-induced increase in TER detected within the first 15 min of treatment [41], and that DN Rac1 expression attenuated S1P-induced increase in TER from baseline in HPAEC [24]. Possible explanations for the differences between the current study and previous reports are the use of different endothelial cell types and experimental conditions [28]. In HUVEC, pretreatment with 10 μM NSC23766 reportedly reduced the increase in barrier function elicited by 0.5 μM S1P [31]. In that study, normalized data were presented, less frequent time points were obtained, and a different detection system (Ussing chamber method) was used to determine TER, which is less sensitive than ECIS [29], With the less frequent time points measured, it is possible that the initial peak increase in TER caused by S1P was missed. It is also notable that because the IC_50_ of NSC23766 is 50 μM that perhaps only a 20% inhibition of Rac1 activity occurred [12]. An additional study, which nicely showed that release caged S1P loaded intracellularly can also increase TER, application of 50 μM NSC23766 could significantly attenuate S1P-induced barrier enhancement [42]. However, it is difficult to compare these results with ours as only one single time point was shown and it is unclear whether NSC23766 had any impact on the baseline barrier function. It is worth noting that we have observed that 50 μM NSC23766 can reduce barrier function of dermal lymphatic endothelial cells and increase permeability of intact, isolated rat mesenteric venules [12]. In current study, we elected to use the Rac1 inhibitor Z62954982 (IC_50_ = 12 μM) that is 4 times more effective than NSC23766 (IC_50_ = 50 μM), which significantly reduced TER, in a concentration-dependent manner. These data indicate reductions in barrier function elicited by inhibition of Rac1 must be taken into consideration in the overall data analysis. Based on the current data, we think that the rapid, S1P-induced, early rise in TER occurs independently of Rac1 activation. The apparent discrepancy of our data from reports in the literature may be summarized by differences in the time points of data collected, whether baseline changes are reported, and perhaps to some extent the endothelial cell types or other experimental conditions. A current limitation is the lack of studies with Rac1 deletion or specific inhibition in intact postcapillary venules, which represents a future step that will help resolve this issue.

Previously we showed that in addition to a transient Rac1 activation, RhoA activity was greatly increased and sustained for at least 10 min upon S1P treatment [12]. In the current study, we employed a FRET biosensor to monitor RhoA activity over time in individual cells. We observed that RhoA activity during our baseline measurements oscillates in the outer peri-nuclear regions. We also observed an overall increase in RhoA activation after S1P treatment, with high levels of RhoA-GTP near endothelial cell borders than in the central areas of cells (Fig 7A). Our results are in agreement with data presented by Szulcek and colleagues, who demonstrated RhoA activation localized near intercellular gaps during their closure [20]. In their study they also demonstrated that RhoA activation in the central area of the cells is barrier disruptive while peripheral RhoA activation is barrier protective. With the concept in mind, it is not surprising that we observed that pretreatment of the endothelial monolayers with RhoA inhibitors attenuated the S1P-induced barrier enhancement that begins almost immediately after S1P is added to the bath. There is some variation of how the Rho inhibitors affect the ability of HUVEC and HDMEC to respond to S1P, which could be due to a variety of reasons including vessel source, donor source, and how well each type of cell thrives in culture. Still, this data suggests that RhoA is involved in the initial rise in TER elicited by S1P, and is in agreement with data from other groups that have shown that inhibition of the RhoA effector, ROCK, attenuates S1P-induced barrier enhancement [10,20,21]. Combined, these data indicate that the RhoA/ROCK pathway contributes, at least in part, to S1P-induced endothelial barrier enhancement. It is also worth noting that in some studies, inhibition of RhoA or ROCK has caused a decrease in the baseline TER [25,43]. Such data supports that the peripheral activation of RhoA indicated by our FRET probe study and that of Szulcek and colleagues [20] contributes to endothelial barrier maintenance.

Several reports have indicated that RhoA- or ROCK-mediated increases in phosphorylation of MLC-2 is endothelial barrier disruptive, particularly with inflammatory stimuli, such as LPS, signals from activated neutrophils, or VEGF [15,16,44–47]. However, Garcia and colleagues characterized that S1P increases cortical MLC-2 phosphorylation [10] and suggested that this contributes to the S1P-induced barrier-protective effect. Moreover, Dudek and colleagues revealed that myosin light chain kinase (MLCK) activation by Abl tyrosine kinase is important for S1P-induced barrier enhancement [11]. Such findings suggested that the role of MLC-2 and the actin cytoskeleton have a general role in mediating either increases or decreases in endothelial barrier function. Concordantly, we observed that S1P significantly increases the phosphorylation of MLC-2 on Ser18/Thr19. Garcia and colleagues also observed that inhibition of MLCK failed to block the ability of S1P to increase TER [10]. With the knowledge that ROCK can increase MLC-2 phosphorylation by inhibiting the MLC-2 phosphatase by phosphorylating the targeting subunit MYPT-1 [9], we studied this alternative pathway. We found that inhibition of RhoA abrogated the S1P-induced phosphorylation of MLC-2 at its regulatory sites. MLC-2 phosphorylation at the cell cortex is thought to stabilize the cortical actin cytoskeleton [23,48]. In addition, myosin activation has been suggested to promote lamellipodia formation [11,49,50], and several reports have suggested that local lamellipodia formation at intercellular junctions contribute to endothelial barrier integrity [12,28,51–54]. We recently showed that S1P increased local lamellipodia at cell borders in association with increased TER, and that blockade of the myosin II ATPase, which selectively reduced local lamellipodia without affecting other actin-containing structures like stress fibers or cortical actin cables, decreased TER [12]. In addition, it is interesting that RhoA inhibition also blocked Vinculin mobilization to the cell periphery. Previous studies have suggested that S1P-induced endothelial barrier enhancement can be VE-cadherin independent[21]. Combined with the data in current study, S1P’s barrier protective effect appears to be complex, as few inhibitors completely blunt its barrier protective effects. Based on our data and those of others [10,11], we think it is reasonable to state that phosphorylation and MLC-2 and actin cytoskeleton activation have general roles in the control of endothelial barrier function, and are likely guided by other factors or by location of action within cells. We speculate that increased MLC-2 phosphorylation at the cell periphery may stabilize cortical actin, promote lamellipodia protrusions that anneal cellular gaps, induce focal adhesion complex assembly and mobilization to the periphery to maintain or enhance endothelial barrier function.

In summary, our results suggest that S1P is able to enhance barrier function independently of Rac1 in HUVEC and HDMEC monolayers. We also presented evidence that S1P activates RhoA primarily near intercellular junctions. Inhibition of RhoA attenuated S1P-induced phosphorylation of MLC-2 near junctions, F-actin assembly and vinculin recruitment at cell periphery, and S1P-induced endothelial barrier enhancement. Our data demonstrate an important role for RhoA-mediated phosphorylation of MLC-2 in S1P-induced endothelial barrier enhancement. Future studies will be needed to elucidate the role of additional RhoA-mediated downstream signals that may underlie S1P-induced endothelial barrier protection.

## Supporting Information

S1 Fig Inhibition of RhoA attenuated S1P-induced barrier enhancement of HUVEC monolayers. A & C. Time courses of TER changes during pretreatment with 25 μM Rhosin (A) or 25 μM Y16 (C), and subsequent treatment with 2 μM S1P or vehicle (N = 8 for each group). B & D. Mean maximal changes in TER (%) within the first 10 min after S1P. *P<0.05, S1P vs. vehicle treated group, same color bar. †P<0.05, inhibitor vs. vehicle pretreatments.
(TIF)

S2 Fig Use of the individual RhoA inhibitors does not attenuate S1P-induced barrier enhancement of HDMEC monolayers. A & C. Time courses of TER changes during pretreatment with 25 μM Rhosin (A) or 25 μM Y16 (C), and subsequent treatment with 2 μM S1P or vehicle (N = 8 for each group). B & D. Mean maximal changes in TER (%) within the first 10 min after S1P. *P<0.05, S1P vs. vehicle treated group, same color bar. †P<0.05, inhibitor vs. vehicle pretreatments.
(TIF)

S1 Movie S1P activated RhoA primarily at cell periphery. This movie shows time lapse images of an endothelial cell expressing the pTriEx-RhoA FLARE.sc Biosensor used to detect RhoA in its GTP-bound, active form. The top panel shows the CFP channel, the middle panel shows the YFP channel, and the bottom panel portrays the YFP:CFP ratio (FRET) indicating RhoA activation. The frames are at 1 min intervals. S1P (2 μM) was added at time = 0 min. The addition caused an obvious shift in GTP-bound RhoA toward the cell periphery in the first minute after the addition of S1P.
(MOV)
