# Supplementary figures and images for: Activation of RhoA, but Not Rac1, Mediates Early Stages of S1P-Induced Endothelial Barrier Enhancement
Source: PLoS One. 2016 May 17;11(5):e0155490. doi: 10.1371/journal.pone.0155490 (PMC4871357; doi:10.1371/journal.pone.0155490)

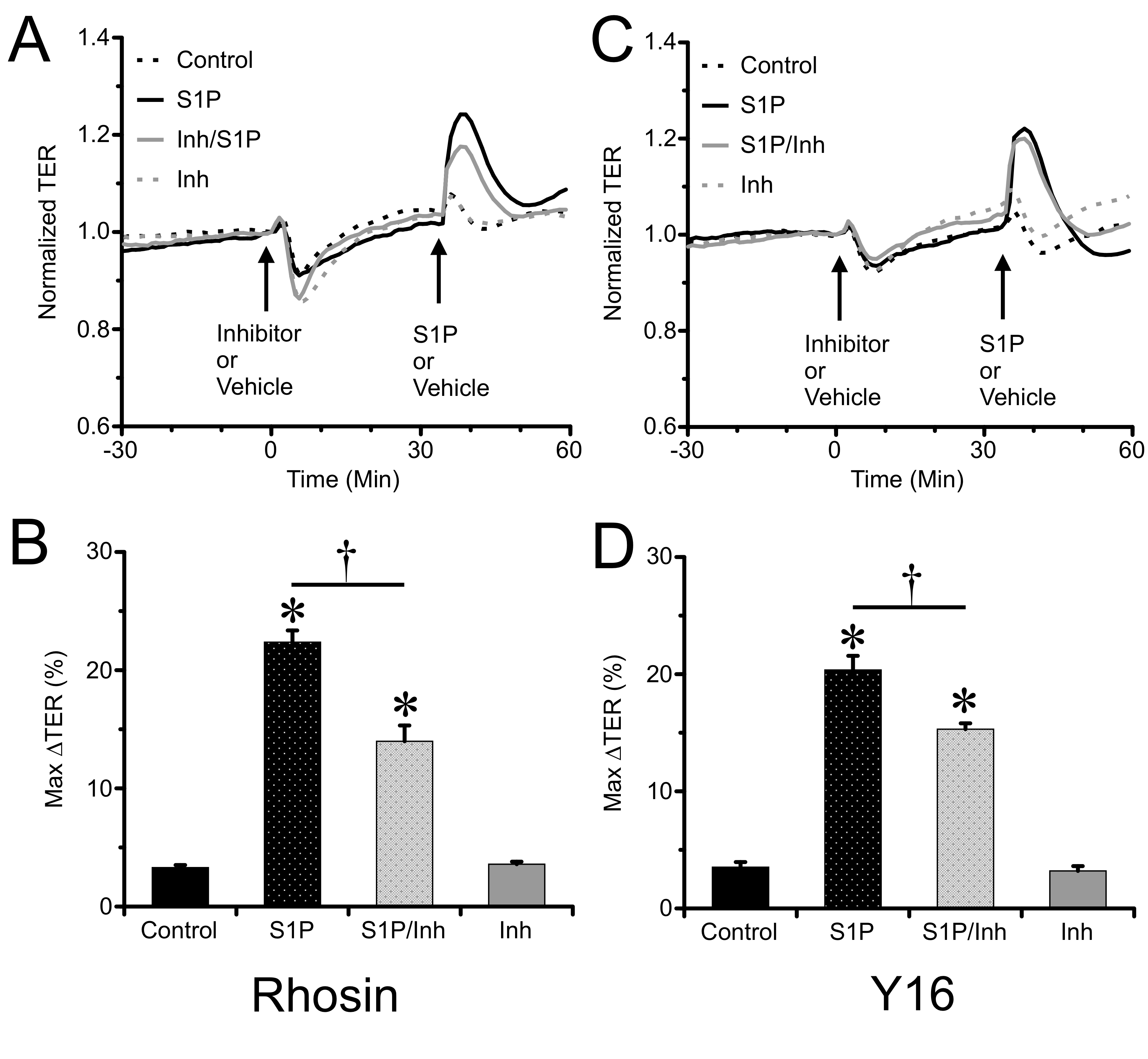

Supplement: S1 Fig — A & C. Time courses of TER changes during pretreatment with 25 μM Rhosin (A) or 25 μM Y16 (C), and subsequent treatment with 2 μM S1P or vehicle (N = 8 for each group). B & D. Mean maximal changes in TER (%) within the first 10 min after S1P. *P<0.05, S1P vs. vehicle treated group, same color bar. †P<0.05, inhibitor vs. vehicle pretreatments. (TIF) [file pone.0155490.s001.tif]

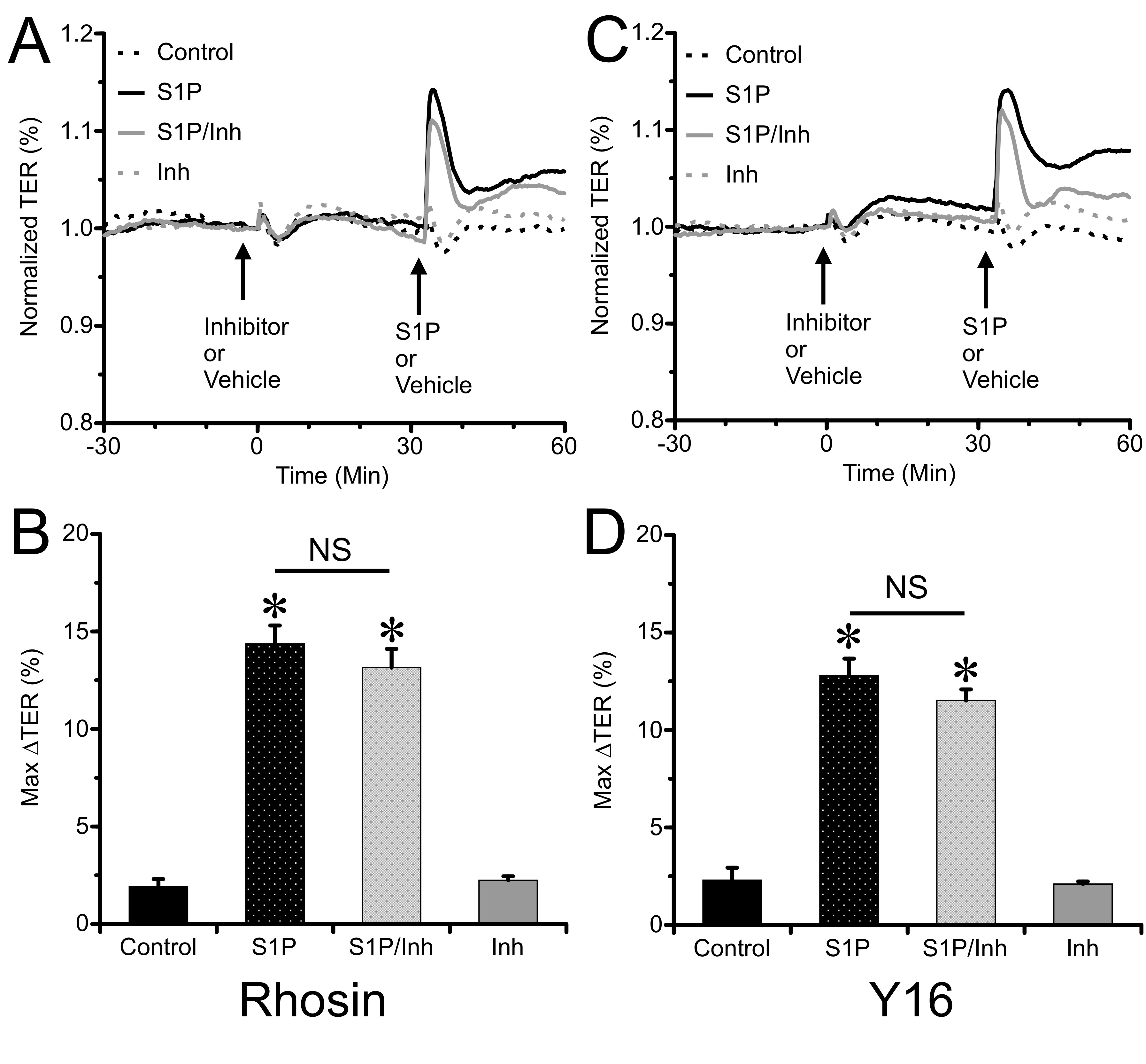

Supplement: S2 Fig — A & C. Time courses of TER changes during pretreatment with 25 μM Rhosin (A) or 25 μM Y16 (C), and subsequent treatment with 2 μM S1P or vehicle (N = 8 for each group). B & D. Mean maximal changes in TER (%) within the first 10 min after S1P. *P<0.05, S1P vs. vehicle treated group, same color bar. †P<0.05, inhibitor vs. vehicle pretreatments. (TIF) [file pone.0155490.s002.tif]
